# Sperm Quality and Tap Water: Disinfection By-Product Effects Not Supported

**DOI:** 10.1289/ehp.115-a416b

**Published:** 2007-08

**Authors:** Valerie J. Brown

Chemicals used to disinfect water often contain chlorine, which can react with organic matter in the water to form compounds such as trihalomethanes (THMs) and haloacetic acids (HAAs), known collectively as disinfection by-products (DBPs). DBP exposure has been implicated in reproductive abnormalities, and earlier human studies have found associations between DBPs and adverse pregnancy outcomes. A new study of DBP exposure now shows only weak evidence of sperm quality damage from exposures at or below regulatory limits **[*EHP* 115:1169–1176; Luben et al.]**.

Earlier studies have revealed little about the degree of risk from DBP exposure, especially for male reproductive health, and there have been few epidemiologic studies of the possible effects on sperm quality. Rodent studies suggest that drinking water exposure to HAAs, particularly brominated species, could pose a threat to human sperm. Because some DBPs are considered carcinogens, there is also concern about DNA damage.

The current study assessed 228 men recruited from couples participating in a project to determine whether DBPs affect spontaneous abortion. The study population was drawn from three locations: one with a water supply containing low overall DBPs, one with low brominated but moderate chlorinated DBPs, and one with low chlorinated but moderate brominated DBPs. “Moderate” was defined as close to but below the U.S. EPA limits for four THMs and five HAAs. The study also analyzed total organic halides (TOX), a group that includes THMs, HAAs, and other organic halides that may not have been identified individually.

Researchers surveyed participants to determine the amount of tap water they ingested and the frequency and length of their showers and baths, then calculated individual DBP exposure estimates. The men also provided semen samples, which the researchers analyzed for total sperm count, sperm maturity and morphology, and DNA integrity.

Expecting more sperm damage at higher DBP exposures, the researchers found instead that the top 25th percentile for both THM and HAA exposure had higher sperm counts than those in the bottom 50th percentile of each group. Results for sperm morphology and DNA integrity were similar. Sperm concentration did decrease as exposure to TOX increased, consistent with findings that TOX may be a greater risk factor for pregnancy difficulties than the individual compounds or groups of compounds now regulated. However, if this were the case, an increase in abnormal sperm morphology with increasing TOX would be expected, and no such increase was observed.

Because the study population was presumed fertile, a small decrease in sperm count might not be detectable against high background counts, and could explain the null results reported by the authors. Because only a few men were exposed to DBPs above regulatory limits, further clarification might be obtained by future studies including a wider range of exposures.

